# Membrane Channels in Health and Diseases

**DOI:** 10.3390/ijms24076719

**Published:** 2023-04-04

**Authors:** Antonio Felipe, Antonio Ferrer-Montiel

**Affiliations:** 1 Red Española de Canales Iónicos (RECI), 03202 Elche, Spain; 2 Molecular Physiology Laboratory, Departament de Bioquímica i Biomedicina Molecular, Institut de Biomedicina (IBUB), Universitat de Barcelona, Avda. Diagonal 643, 08028 Barcelona, Spain; 3 Instituto de Investigación, Desarrollo e Innovación en Biotecnología Sanitaria de Elche (IDiBE), Universitas Miguel Hernández, Av. de la Universidad s/n, 03202 Elche, Spain

The goal of this Special Issue, entitled “Membrane Channels in Health and Diseases (https://www.mdpi.com/journal/ijms/special_issues/Membrane_Channels_Health_Diseases; accessed on 18 March 2023)”, is to collect new and updated ion channel contributions from the Spanish Ion Channel Network (Red Española de Canales Iónicos, RECI; https://www.reci-ionchannel.es/; accessed on 18 March 2023) and worldwide colleagues. This Special Issue is the second, updated edition of the first compilation, “Membrane Channels in Human Diseases”, launched in 2018, which was successful in terms of global visibility and acceptance. In fact, by the time this editorial is published, a third edition, “Membrane Channels in Physiology and Pathology”, a Special Issue included in the section “Molecular Biology” of *IJMS*, will be on its way. This second edition hosts 17 contributions, 13 original research articles and 4 review papers, and has been even more successful in terms of worldwide visibility, being viewed more than 33,000 times at the time of writing.

Ion channels are crucial components of the cellular machinery, conducting ions across cell membranes. These proteins generate and maintain the electrical potential across cell membranes, being essential for a wide range of biological processes, including nerve conduction, muscle contraction and the immune system response, among other physiological events. Ion channels are proteins that span the lipid bilayer of the cell membrane, forming a selective pore. The channel’s structure determines the ion selectivity via a filter that interacts with ions passing through the pore. The opening and closing of channels are tightly regulated, allowing cells to respond rapidly to changes in their environment. While some ion channels depend on voltage, functioning in response to changes in the membrane potential, others are activated by ligands, such as neurotransmitters or hormones that bind to the channel, triggering downstream events. Ion channel malfunctions are implicated in a wide range of diseases (channelopathies), including cystic fibrosis, epilepsy, cardiac arrhythmias and cancer. Therefore, drugs targeting ion channels are useful as therapeutic agents. The study of ion channels is constantly evolving based on advances in electrophysiology, biochemistry and genetics. New advances in X-ray crystallography and cryo-electron microscopy push forward the knowledge of their protein structure, providing insights about function and regulation.

In this evolving scenario, our Special Issue, “Membrane Channels in Health and Diseases”, compiles new and exciting works that advance our understanding of ion channels and how these proteins contribute to fundamental cellular mechanisms, opening up new avenues for therapeutic interventions. This Special Issue brings together a collection of articles from researchers around the world, displaying the latest research and developments in the field of membrane channels and their contributions to health and disease. The published articles cover a wide range of topics related to membrane channels, including ion channels, receptors and transporters. Many articles explore the role of membrane channels in various pathologies, such as cancer, neurological disorders and cardiovascular disease. In addition, new and exciting contributions deepen our understanding of complementary interventions, such as machine learning and optogenetics. Bearing all this in mind, we, the editors of this Special Issue, thank the contributors for their effort and hope that the reading of these selected contributions will be enjoyable.

